# Stability of Plasmonic Mg-MgO Core–Shell Nanoparticles in Gas-Phase Oxidative Environments

**DOI:** 10.1021/acs.nanolett.4c01720

**Published:** 2024-05-30

**Authors:** Vladimir Lomonosov, Jinfeng Yang, Ye Fan, Stephan Hofmann, Emilie Ringe

**Affiliations:** † Department of Materials Science and Metallurgy, University of Cambridge, 27 Charles Babbage Road, Cambridge CB3 0FS, United Kingdom; ‡ Department of Earth Sciences, University of Cambridge, Downing Street, Cambridge CB2 3EQ, United Kingdom; § Department of Engineering, University of Cambridge, Cambridge CB3 0FA, U.K.

**Keywords:** Magnesium nanoparticles, nanoparticle stability, oxidation, characterization *in situ*, localized surface plasmon resonance, *in situ* SEM, plasmon-enhanced catalysis

## Abstract

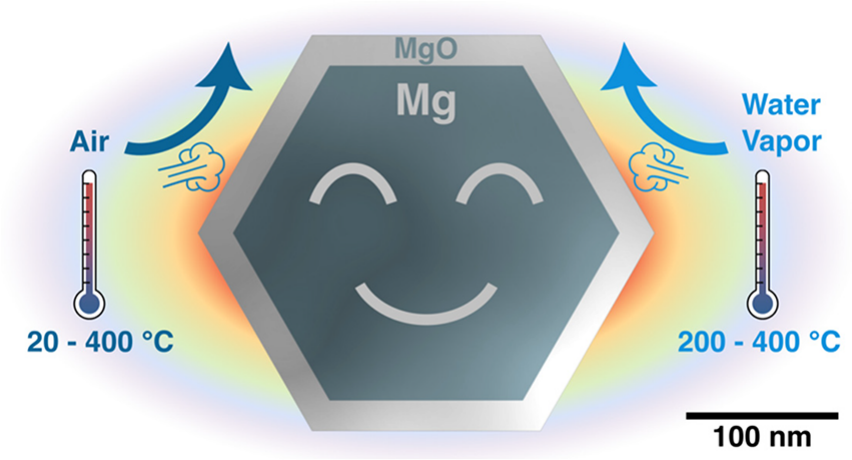

Magnesium is a recent addition to the plasmonic toolbox: nanomaterials that efficiently utilize photons’ energy due to their ability to sustain localized surface plasmon resonances. Magnesium nanoparticles protected by a native oxide shell can efficiently absorb light across the solar spectrum, making them a promising photocatalytic material. However, their inherent reactivity toward oxidation may limit the number of reactions in which Mg-MgO can be used. Here, we investigate the stability of plasmonic Mg-MgO core–shell nanoplates under oxidative conditions. We demonstrate that the MgO shell stabilizes the metallic Mg core against oxidation in air at up to 400 °C. Furthermore, we show that the reactivity of Mg-MgO nanoplates with water vapor (3.5 vol % in N_2_) decreases with temperature, with no oxidation of the Mg core detected from 200 to 400 °C. This work unravels the potential of Mg-MgO nanoparticles for a broad range of catalytic transformations occurring in oxidative environments.

Magnesium (Mg), the eighth most abundant element in Earth’s crust, is attractive for energy conversion,^1,2^ energy storage,^3−5^ and chemical synthesis.^6,7^ In recent years, Mg nanoparticles have drawn growing attention as an alternative to established plasmonic metals, such as gold, silver, and copper. In addition to its abundance and biocompatibility,^8−10^ Mg is characterized by an exceptional potential for harnessing solar energy due its ability to sustain localized surface plasmon resonances (LSPRs) across the UV–visible-NIR wavelengths, i.e., the entire solar spectrum.^11−13^ However, Mg is also known for its high reactivity, in particular, toward oxidation, and ability to ignite at a relatively low temperature. Indeed, Mg ribbons burning in air or reacting with CO_2_ in a thermite-like reaction^14^ produce a dazzling white flame often used as a chemical demonstration in school that can leave a strong impression related to the reactivity and safety of Mg metal.

Fortunately, Mg metal oxidizes spontaneously in air at room temperature to form a thin self-limiting MgO layer that inhibits further reactivity in ambient conditions.^13,15^ The formation of this protective oxide can be explained by the coupled currents approach,^16,17^ where solid-state, outward diffusion of cations or inward diffusion of anions is balanced by the transport of electrons from the metal-oxide interface. Since tunneling, the only electron transport mechanism available at room temperature, decreases exponentially with oxide layer thickness, the oxide growth essentially stops after its thickness reaches a few nanometers. For Mg, the Mg^2+^ cations diffuse, and as the oxide grows, transport of cations and electrons becomes increasingly unlikely, leading to the experimentally observed parabolic growth kinetics of the oxide layer.^18^ Stabilization approaches aiming to change the composition and hence growth properties of the surface layer are well-developed for bulk Mg. These include alloying, where additives such as Al, Be, and Ca, to name only a few, modify the oxide growth and composition,^18−22^ as well as surface treatments such as with CO_2_ plasma.^23^ At present, neither of these stabilization strategies have been reported for nanoplates (NPs) of Mg, such that this paper focuses on the intrinsic stability of Mg-MgO NPs.

Mg NP’s native ≤10 nm oxide shell imparts stability without being detrimental to plasmonic properties such as light absorption and scattering of the Mg core.^12,13,24^ Furthermore, due to near-field effects, oxide-coated plasmonic structures decorated with a catalytically active, albeit poorly plasmonic metal, can demonstrate strongly enhanced photocatalytic performance. For instance, Swearer et al.^25^ showed that the plasmon-enhanced electromagnetic field at the surface of Al nanoparticles covered with a native oxide layer can lead to a drastic increase in hot carrier production in the Pd islands decorating the surface, despite the Al and Pd being separated by the aluminum oxide. We further demonstrated that Mg-MgO core–shell nanoparticles decorated with Au^26^ and Pd^27^ exhibit excellent photocatalytic behavior in the coupling of 4-nitrobenzenethiol and selective hydrogenation of acetylene, respectively, again despite the presence of an ∼10 nm oxide layer. In the latter, an over 2-fold decrease in activation energy under light excitation compared to the dark, thermally driven reaction was obtained, together with a stable catalytic performance over at least 4 h.

But can Mg-MgO core–shell nanostructures be used to choreograph a large range of reactions, including those that proceed in an oxidative environment and/or generate products that can act as oxidizing agents (*e.g*., water vapor)? To address this question, one needs to understand how protective the native oxide is in catalytically relevant oxidative environments. The answer we reveal destigmatizes Mg, shifting its image from that of a highly reactive material to the reality in which the oxide layer provides significant protection across a broad range of environments relevant for catalysis.

Specifically, we investigated the long-term stability of plasmonic Mg-MgO nanoplates (NPs) in ambient conditions and their oxidation by air and water vapor across a wide temperature interval (20–550 °C) below Mg’s melting point (650 °C). We show that the stability of plasmonic Mg cores conferred by the MgO shells under conditions (temperatures and environments) relevant to gas-phase heterogeneous catalysis can extend their application far beyond ambient and reductive conditions. This work and its findings aim to encourage the scientific community to turn its attention to sustainable plasmonic Mg-MgO nanostructures and explore such compositions in a variety of catalytic transformations that can benefit from plasmon-enhanced activation. Mg-MgO core–shell NPs were synthesized via a modified, previously reported^28^ colloidal seed-mediated synthesis (full methods in Supporting Information). The resulting NPs (Figures 1a, S1) comprise a mixture of single-crystalline hexagonal plates (mean ± standard deviation, 250 ± 30 nm) and singly twinned folded plates (310 ± 60 nm), with a native oxide layer described in detail elsewhere.^12,13,24,29,30^

**Figure 1:**
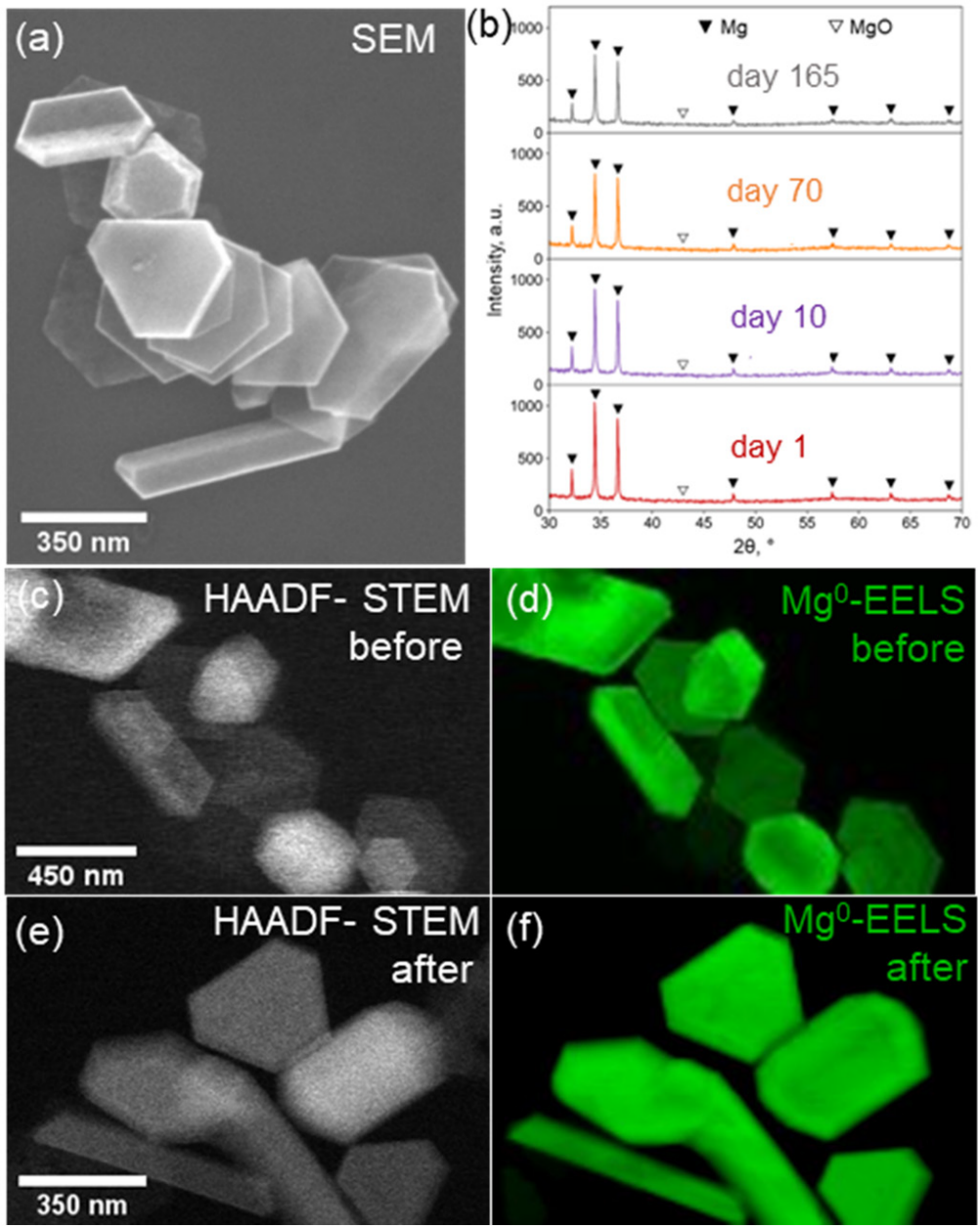
Stability of Mg-MgO NPs in ambient air and oxygen plasma. (a) Representative SEM image of as-prepared Mg-MgO NPs; (b) powder XRD patterns of Mg-MgO NPs stored in ambient air for 1 day (red line), 10 days (purple line), 70 days (orange line), and 165 days (gray line); (c, e) HAADF-STEM images and (d, f) associated STEM-EELS maps of the bulk Mg plasmon indicative of metallic Mg for Mg-MgO NPs before and after treatment in oxygen plasma (25% O_2_ in Ar, 13.56 MHz, 5 min).

## Stability in Ambient Conditions

First, we investigated the long-term stability of Mg-MgO NPs in ambient air to assess their storage requirements. 5 μL of Mg-MgO colloid in isopropanol (2.5 mg/mL) was drop-cast on a Si wafer and left to dry in air at room temperature for 1 h. The X-ray diffraction (XRD) pattern of the resulting finely spread powder revealed peaks corresponding to metallic Mg’s hexagonal close-packed crystal structure (JCPDS 04-0770). No MgO (JCPDS 89-7746) peaks were observed (Figure 1b), indicating that the oxide shell has a small domain size, consistent with a polycrystalline, sub-10 nm shell, and in good agreement with the XRD data for Mg-MgO NPs reported previously.^13^ The Mg-MgO sample was left in ambient air (20 °C and 50–60% humidity), and XRD analysis was performed periodically over 165 days. No change in the metallic Mg signal or evolution of MgO signal was detected over this period, confirming that the MgO shell is protective and retained its initial structure (Figure 1b).

Inductively coupled radio frequency oxygen plasma offers a more reactive environment than that of ambient air. In addition to being a common step for dislodging hydrocarbons, it also forms ions and radicals that are common reaction intermediates in the catalytic utilization of light alkanes including methane and carbon dioxide.^31,32^ Scanning transmission electron microscopy (STEM) and STEM-electron energy loss spectroscopy (STEM-EELS) of Mg-MgO NPs treated with oxygen plasma (25% O_2_ in Ar, 13.56 MHz, 5 min) confirmed that neither the morphology of the NPs nor the metallic character of their cores (as revealed by the bulk plasmon signal of metallic Mg) were affected by oxygen plasma exposure (Figures 1c-f, S2).

## High-Temperature Oxidation in Air

At high temperatures, diffusion rates increase, and electron transfer via thermal emission becomes possible. Therefore, faster oxidation kinetics is anticipated. We used an array of approaches including thermogravimetric analysis (TGA), *in situ* XRD, and *in situ* scanning electron microscopy (SEM) to investigate the oxidation dynamics of Mg-MgO NPs across a temperature interval of 20–550 °C.

**Figure 2:**
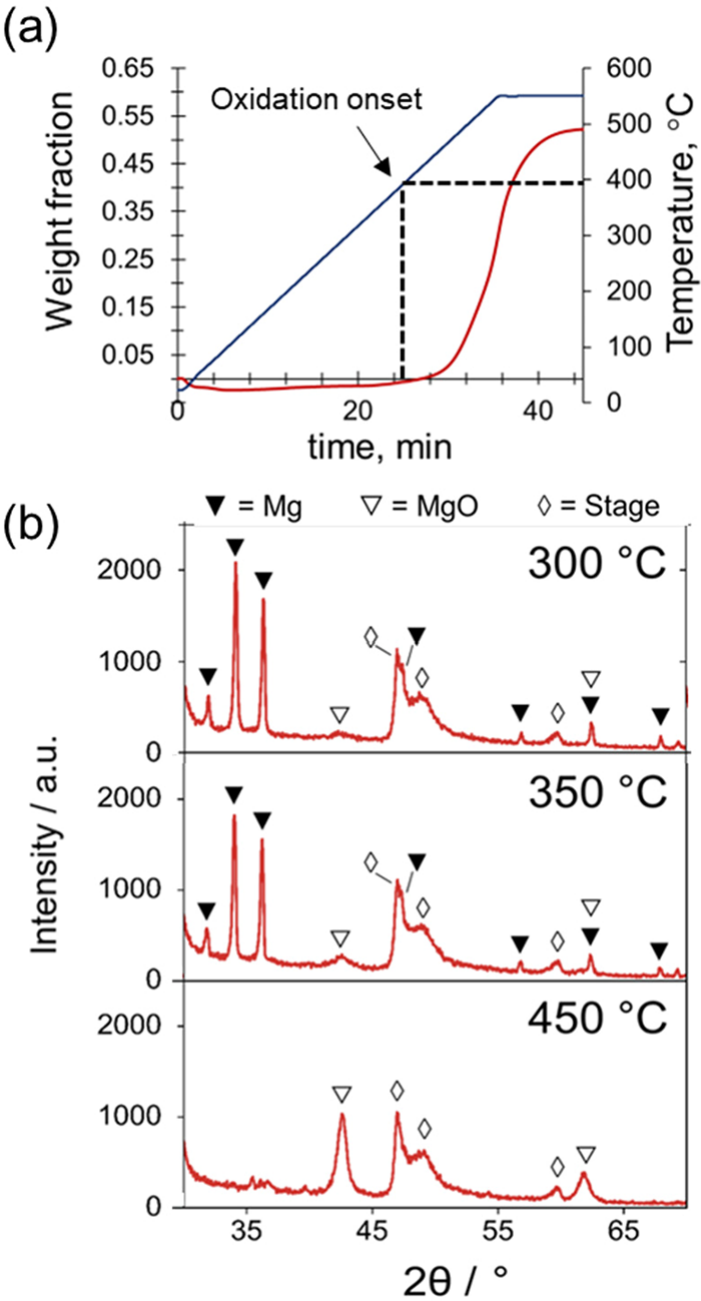
Oxidation behavior of Mg-MgO NPs powders in air across the temperature interval 20–550 °C. (a) Thermogravimetric analysis performed at 15 °C/min; (b) *in situ* XRD with the sample stepwise heated 10 °C/min in air from room temperature to 450 °C in increments of 50 °C.

For TGA, 5 mg of Mg-MgO NPs powder was heated in air (50% humidity) from room temperature to 550 °C at 15 °C/min and kept at this temperature for 20 min. No noticeable change in the sample mass was detected below 380 °C (Figure 2a), indicating the absence of further oxidation. The oxidation onset at 380 °C is characterized by a rapid increase in the sample mass, which completes at 550 °C. The total mass increase at the end of the experiment was equal to 52.2%, slightly below the expected stoichiometric value for oxidation of Mg to MgO (65.8%). This discrepancy is explained by the presence of an oxide shell in the initial sample. These results are in a good agreement with TGA data reported by other groups for micron-sized Mg powders^33−35^ and Mg NPs synthesized by a vapor-phase method.^1^

The stability of metallic Mg in Mg-MgO NPs in air (50% humidity) up to 400 °C is supported by *in situ* XRD. A finely spread powder of Mg-MgO NPs on a Si wafer was stepwise heated in air in an XRD characterization chamber from room temperature to 450 °C, at 10 °C/min in increments of 50 °C. The sample was maintained under isothermal conditions for 20 min prior to each XRD analysis. No oxidation was detected at temperatures below 300 °C (Figures 2b, S3). However, a small peak corresponding to MgO evolves between 300 and 350 °C and becomes more pronounced at 400 °C. Importantly, no obvious change in the intensity of the Mg peaks was observed, indicating that the sample remained metallic. The XRD pattern changes drastically at 450 °C: MgO is the only detected phase, and no signs of metallic Mg are left, indicating complete oxidation. The good correlation between TGA and XRD confirms the stability of the Mg core against oxidation in air for the temperature interval of 20–400 °C.

**Figure 3:**
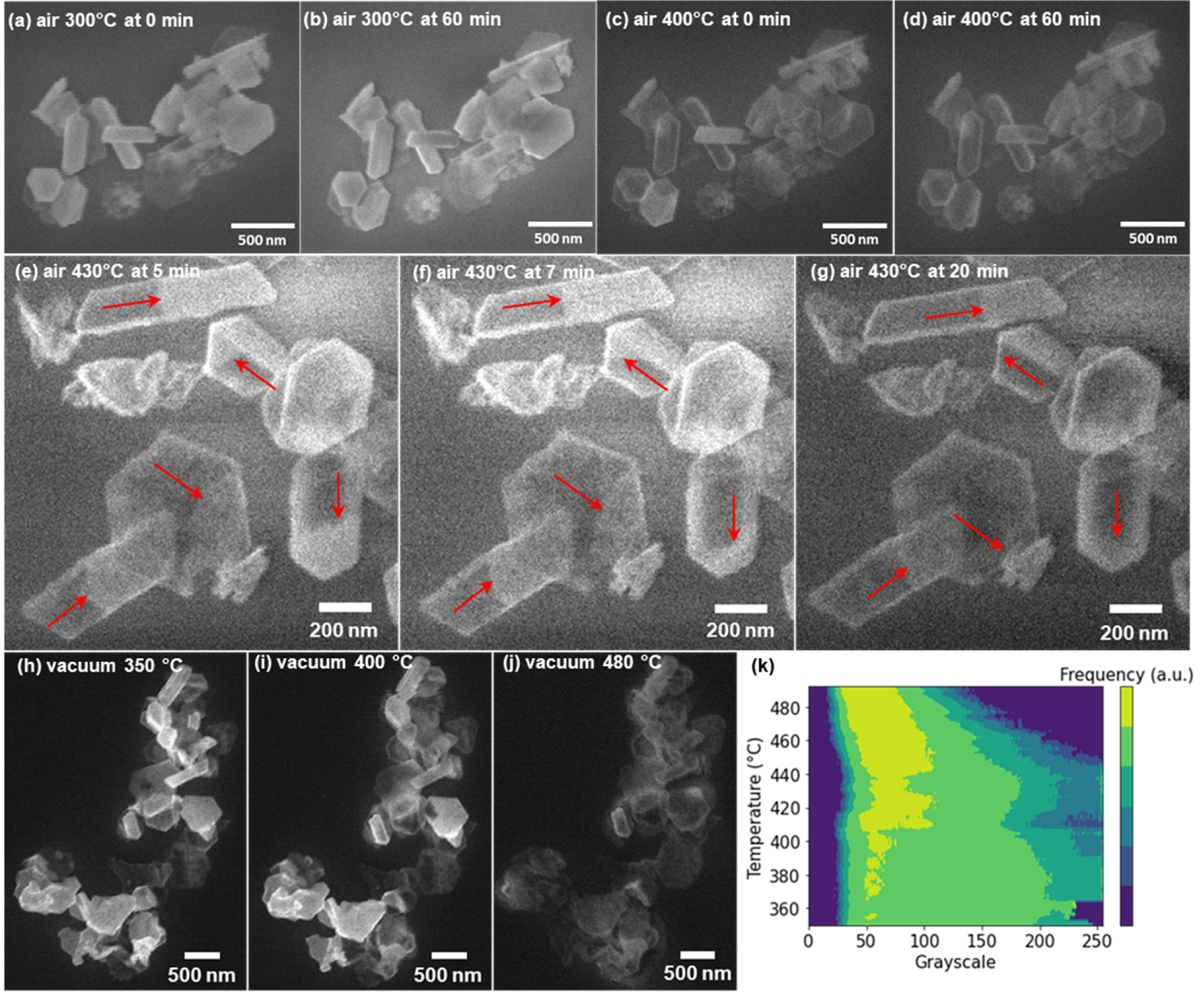
Thermal treatment of Mg-MgO NPs in air and a vacuum. *In-situ* SEM images of Mg-MgO NPs exposed to ∼100 mbar of air at (a, b) 300 °C and (c, d) 400 °C taken after 0 and 60 min of exposure. *In-situ* SEM images of Mg-MgO NPs exposed to ∼500 mbar of air at 430 °C were taken after (e) 5, (f) 7, and (g) 20 min of exposure. The arrows indicate the progression of the hollowing of NPs. *In-situ* SEM images of Mg-MgO NPs heated in vacuum (10^–6^ mbar) to (h) 350, (i) 400, and (j) 480 °C. (k) Histogram of the distribution of grayscale in the images of the same region acquired at different temperatures in a vacuum. Additional images reported in Figure S5.

In order to evaluate potential macroscale effects such as heat and mass transfer in bulk powders, we studied the oxidation of Mg-MgO NPs for isolated NPs with *in situ* SEM. Mg-MgO NPs dispersed on a Si wafer were locally exposed to ∼100 mbar of air at 300–430 °C using a customized setup that allows high-resolution SE imaging (Supporting Information Methods and Figure S4). After 1 h of air exposure at 300 °C, no change in NP morphology or contrast was observed (Figure 3a,b). However, when the temperature was increased to 400 °C, faceted nanovoids were formed (Figure 3c), some leading to fully hollow structures after 1 h of exposure (Figure 3d).

To further investigate the dynamics of the hollowing process, a Mg-MgO sample dispersed on a Si wafer was heated to 430 °C and exposed to 100 mbar of air, and SEM images were acquired *in situ* after 5, 7, and 20 min of exposure. Continuous unidirectional hollowing was observed, indicating the anisotropic character of the oxidation process (Figure 3e-g, Supporting Information video 1).

Anisotropic oxidative hollowing of Mg NPs was previously observed at 340–350 °C by *in situ* TEM^1,2^ and explained by the high vapor pressure of Mg. It was suggested that the high volatility of Mg facilitates its outward diffusion through the oxide shell, and that oxidation can be initiated in the vapor phase.^1,2^ Such oxidation behavior resembles the formation of hollow nanostructures of cobalt oxide and sulfide through the nanoscale Kirkendall effect.^36^ However, in our study, the hollowing of Mg-MgO is not accompanied by an increase of the size of the NPs (Figures 3, S5) indicating the absence of outward oxide growth. This sublimation-enhanced oxidation of Mg nonaparticles was observed by *in situ* TEM at substantially lower temperatures (∼200 °C) by Zhang et al.^37^ for arc plasma deposited nanoparticles. We attribute their lower oxidation onset temperature to the contribution of the continuous exposure of Mg nanoparticles to a high-energy electron beam; we confirm this explanation via our observation of hollowing for Mg-MgO NPs at 250 °C upon continuous SEM electron beam irradiation (Supporting Information video 2).

To further investigate the sublimation-induced hollowing of Mg-MgO NPs we heated them at 5 °C/min in an SEM chamber under an inert Ar atmosphere at a pressure 10^–6^ mbar. In these images (Figure 3h-j, Supporting Information video 3), hollowing or thinning of the NPs leads to a decrease in secondary electron intensity, ultimately approaching that of the substrate. Given that the images were acquired sequentially and under the same conditions except for temperature, the secondary electron intensity can be approximated by the grayscale value (additional processing details in the Supporting Information). A gradual hollowing was observed in vacuum at temperatures above 400 °C, as evidenced by the overall darkening of the NP-containing regions from 350 to 400 to 480 °C (Figure 3h-j, Supporting Information video 3). To further evidence this transition, we plotted the grayscale distribution for each temperature in Figure 3k; a transition clearly occurs above 400 °C when lower grayscale values become more common across the image, indicating a thinning of the Mg layer associated with hollowing.^1^

While the protective behavior of the oxide layer is generally explained by a diffusion-controlled process, the absence of oxidation followed by a very rapid oxidation observed for Mg-MgO bulk powder (Figure 2) and hollowing of single particles (Figure 3) at temperatures above 400 °C indicates a change in the mechanism. A growing, thick MgO layer was previously suggested to fail at protecting the underlying Mg due to pores and cracks formation attributed to the volume mismatch between Mg and MgO (Pilling–Bedworth ratio of 0.81).^33^ However, MgO grows extremely slowly even at elevated temperatures: its experimental growth kinetics at 300 °C follows an inverse logarithmic law,^38^ while kinetic modeling of the diffusion-limited oxidation of Mg demonstrated growth from 10 to 30 nm thick MgO in 14 h at 400 °C and 0.21 atm O_2_.^39^ Such slow growth is unlikely to produce the sudden, fast oxidation/hollowing we observe, and instead, we attribute this behavior to the high vapor pressure of Mg at 400 °C and above, leading to its enhanced penetration through the oxide shell. Additionally, as suggested by Ghildiyal et al.,^1^ cracks and pores are likely formed not by the Mg-MgO volume mismatch but rather by the partial vaporization of the Mg core, which causes a pressure buildup and tensile stresses. This vaporization-driven process is further supported by the Mg-MgO hollowing even in the absence of oxygen (Figure 3h-k).

The high-temperature oxidation of Mg-MgO NPs in air revealed a substantial difference in oxidation behavior between dried powders and isolated NPs. Particularly, the oxidation of the bulk Mg-MgO powders results in the formation of crystalline MgO, as confirmed by XRD analysis and by a mass increase (TGA) indicating that most of the Mg was converted to MgO. In contrast, heating of isolated NPs (*in situ* SEM) both in air and vacuum leads to the formation of hollow MgO-confined structures without detectable increase in the oxide thickness. This difference is attributed to mass transport limitations in powders resulting in accumulation of the MgO phase in the sample, whereas for isolated NPs (*in situ* SEM), the produced MgO can quickly dissipate in the SEM chamber, leaving a hollow structure. One commonality, however, is that both bulk and single-particle studies confirm that the MgO oxide shell provides stability to Mg cores toward oxidation in air at temperatures up to 400 °C.

## Gas-Phase Oxidation in Humid Environments

**Figure 4:**
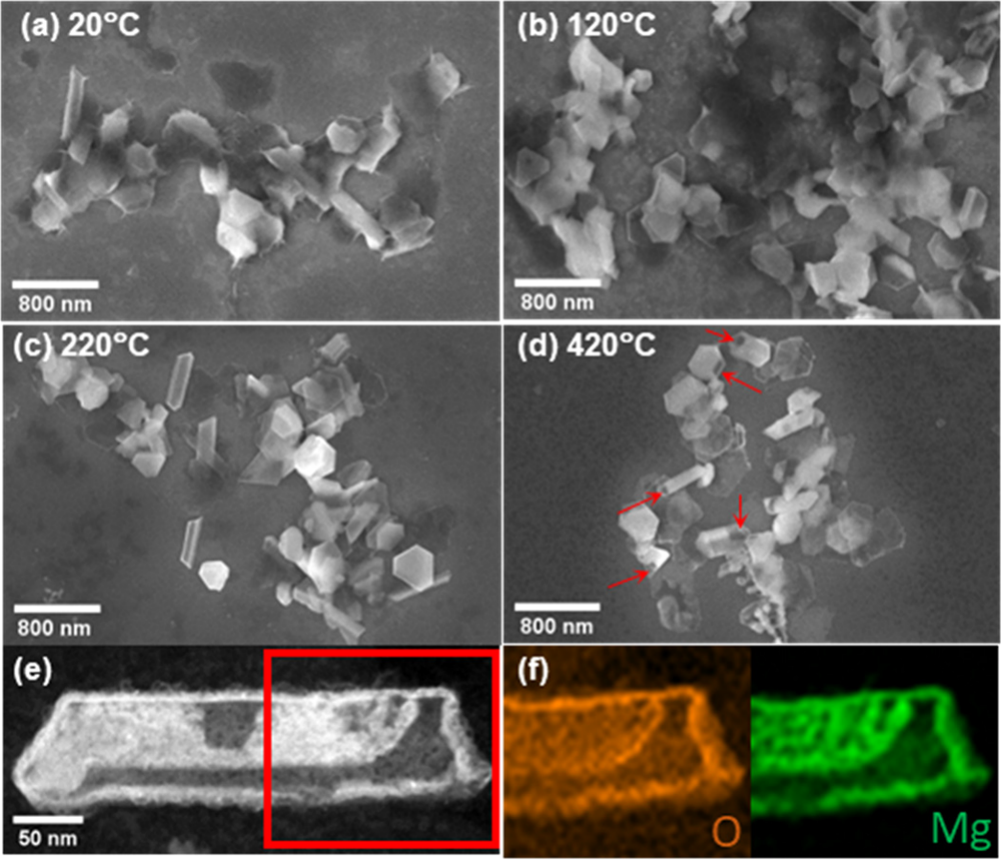
Oxidation behavior of Mg-MgO NPs in humid nitrogen across the temperature interval 20–420 °C. (a–d) SEM images of Mg-MgO NPs treated with 3.5 vol % of H_2_O in N_2_ for 1 h at 20, 120, 220, and 420 °C; (e) HAADF-STEM image and (f) associated STEM-EDS maps of Mg and O of an Mg-MgO NP treated with 3.5 vol % of H_2_O in N_2_ for 1 h at 420 °C. Additional images reported in Figure S6; additional EDS map in Figure S7.

Water, a common byproduct in industrially relevant gas-phase catalytic reactions, is known to corrode Mg due to the formation of non-self-limiting magnesium hydroxide (Mg(OH)_2_). Although the stability of Mg-MgO NPs in aqueous conditions can be extended by encapsulation in a protective 20–30 nm polydopamine shell,^40^ this additional polymer coating on top of a native oxide layer can become detrimental to photocatalytic applications. The oxidation of micron-sized Mg powders in humid argon and water steam at low (40–80 °C) and high (350–650 °C) temperatures was recently reported.^41^ It was found that the activation energy of Mg oxidation in humid environments increased from 60 kJ/mol at low temperatures to 360 kJ/mol at high temperatures. Importantly, while at low temperatures Mg was prone to corrosion in a humid environment, the oxidation onset at high temperatures was found to be above 450 °C.^41^ Here, we studied the intermediate, previously unexplored temperature range of 20–420 °C to reveal the behavior of Mg-MgO NPs, drop-cast on Si wafers, toward water (3.5 vol % water vapor in nitrogen). The samples were first heated in dry N_2_, then purged with the humid N_2_ for 1 h at the selected temperature under isothermal conditions, and finally cooled in dry N_2_ and imaged in an SEM. The oxidation of Mg-MgO NPs with water at low temperatures (20–80 °C) is accompanied by a drastic change in morphology: the initially sharp facets of Mg-MgO NPs become fuzzy with randomly located spikes across the edges of the NPs (Figures 4a, S5). Here, water likely interacts with the oxide shell to form a low-density magnesium hydroxide. Furthermore, formation of Mg(OH)_2_ within the oxide could lead to mechanical stresses which can result in shell rupture.^42^ The effect of water on Mg-MgO NPs morphology becomes less pronounced at 120 °C, whereas no change in the NPs morphology was observed between 220 and 420 °C (Figure 4b-d).

Three factors are responsible for the stability window in water vapor: a diffusion barrier formed by a dense oxide shell, the reduced adsorption of water at elevated temperatures, and the thermodynamic instability of Mg(OH)_2_ at high temperatures. Indeed, Mg(OH)_2_ forms from the reaction of MgO and adsorbed water, and the extent of adsorption strongly depends on the water partial pressure and temperature. Razouk and Mikhail^43^ concluded that the stoichiometric uptake of water by a MgO surface occurs only at saturated vapor pressure at 35 °C and that below this level only some equilibrium amount of water can adsorb. Further, Bratton and Brindley^44^ found that partial coverage of the MgO surface with water molecules greatly suppresses nucleation and almost inhibits the reaction. As temperature increases, one expects the rate constant for the reaction between MgO and water to increase following Arrhenius law; however, the reaction rate is limited by the availability of water owing to decreased adsorption. For instance, a near-zero reaction rate between MgO powders and water vapor at 98 °C was observed at water pressures below 0.19 atm due to insufficient adsorption of water at this temperature.^45^ This correlates well with our observations of only a small effect of water vapor on the morphology of Mg-MgO platelets at 120 °C and no effect at temperatures up to 420 °C. Further, at temperatures above 300 °C the formation of Mg(OH)_2_ from MgO becomes thermodynamically unfavorable.^46^

At temperatures above 420 °C, exposing Mg-MgO NPs to humid N_2_ leads to the formation of faceted voids, as observed in high-temperature air or vacuum (Figures 3,4). STEM-EDS also reveals that these NPs comprise a partially hollow MgO shell and a metallic Mg core (Figure 4d), confirming the sublimation-driven process discussed above. These results indicate that Mg-MgO NPs can resist oxidation in moderately humid environments across the temperature range 200–400 °C; however, the extent of this temperature window can be affected by other parameters such as water vapor concentration and reaction pressure as well as the presence of reaction-specific species such as oxidizing agents or free radicals.

In conclusion, our results demonstrate the stability of plasmonic Mg-MgO core–shell NPs toward oxidation in air at temperatures from room temperature up to 400 °C and with water vapor between 200 and 400 °C. These results were obtained via multiple approaches, including *in situ* oxidation studies and a variety of electron microscopy techniques. While some differences were observed between studies on isolated NPs and bulk methods, the results across TGA, XRD, and SEM reveal a consistent stability window which has the potential to expand the application range of Mg-MgO nanoparticles in plasmon-enhanced catalysis.

## Supporting Information Available

Continuous unidirectional hollowing (AVI)
Hollowing for Mg-MgO NPs at 250 °C upon continuous SEM electron beam irradiation (AVI)
Gradual hollowing observed in vacuum at temperatures above 400 °C, as evidenced by the overall darkening of the NP-containing regions from 350 to 400 to 480 °C (AVI)
Details of Mg-MgO NPs synthesis, TGA, XRD, *in situ* SEM, HAADF-TEM, SEM data processing, oxidation experiments, and additional XRD data and SEM images (PDF)

## Supplementary Material

nl4c01720_si_001.avi

nl4c01720_si_002.avi

nl4c01720_si_003.avi
