# Supplementary material for: Stability of Plasmonic Mg-MgO Core–Shell Nanoparticles in Gas-Phase Oxidative Environments
Source: Nano Lett. 2024 May 30;24(23):7084–90. doi: 10.1021/acs.nanolett.4c01720 (PMC11177309; doi:10.1021/acs.nanolett.4c01720)
Supplement: Supplementary file 4 — nl4c01720_si_004.pdf [file nl4c01720_si_004.pdf]

Supporting Information for

Stability of Plasmonic Mg-MgO Core-shell Nanoparticles in Gas-phase Oxidative Environments

Vladimir Lomonosov,[1,2] Jinfeng Yang[3], Ye Fan,[3] Stephan Hofmann,[3] and Emilie Ringe[1,2]*

1. Department of Materials Science and Metallurgy, University of Cambridge, 27 Charles Babbage Road, Cambridge, United Kingdom, CB3 0FS, United Kingdom
2. Department of Earth Sciences, University of Cambridge, Downing Street, Cambridge, United Kingdom, CB2 3EQ, United Kingdom
3. Department of Engineering, University of Cambridge, Cambridge CB3 0FA, UK.

* Corresponding author: er407@cam.ac.uk; +44 (0)1223 334330 (ph.), +44 (0)1223 334567 (fax).

Methods

Materials

Li pellets (99%), naphthalene, 0.7M n-Butyl-sec-butylmagnesium ($C_8H_{18}Mg$) solution in hexane, poly(vinyl pyrrolidone) (PVP, MW 10,000), anhydrous tetrahydrofuran (THF) and anhydrous isopropanol (IPA) were purchased from Sigma-Aldrich and used as supplied. All glassware was washed with nitric acid and flame-dried under vacuum before use.

Mg-MgO NP synthesis

Mg nano-platelets were synthesized via two-step reduction of $C_8H_{18}Mg$ using Li_2Napht as reducing agent. Li_2Napht solution characterized by deep purple colour was synthesized as follows: 0.028 g of Li pellets (4.05 mmol), 0.260 g of dry naphthalene (2.03 mmol), 0.02 g PVP (0.18 mmol monomer) and 10.75 mL of degassed anhydrous THF were added to a 25 mL Schlenk flask under Ar atmosphere and sonicated for 45 minutes (Allendale Ultrasonics, 100 W 3 L). Mg NP synthesis was initiated by injecting 2 mL of $C_8H_{18}Mg$ into freshly prepared Li_2Napht solution followed by addition of 2 mL of naphthalene in THF (1.0 M) after 5 minutes of reaction, to convert all unreacted Li_2Napht to LiNapht. The resulting mixture was left to react for 60 minutes and then quenched with 2 mL of IPA. The solid product was recovered by centrifugation and residual by-products were removed by centrifugation and redispersion steps in anhydrous IPA twice, anhydrous THF twice and anhydrous IPA twice, before redispersing in anhydrous IPA.

Characterization

***In-situ* Scanning Electron microscopy (SEM)** was performed on Zeiss GeminiSEM 300 equipped with in-lens detector and with a Kammrath & Weiss 1050°C Heating Module. The SEM images were acquired with the electron beam voltage 5 kV, the aperture size 30 µm, and 13mm working distance. The ramping rate of the heater was 20°C/min. The temperature measurement of the heating module was calibrated by the melting point of Na_2WO_4 particles (~100 µm) placed on a Si chip with 200 nm oxide layer. The melting point of Na_2WO_4 was set to be 698°C, and the measurement between 20°C and 698°C was assumed to be linear.

Air was introduced locally to the sample through a tapered quartz nozzle (Figure S4a), made by a P-2000 Laser Based Micropipette Puller. The quartz nozzle has an opening with a diameter of 9.6 μm, and is 10 μm above the substrate, 30° with respect to the substrate (schematically shown in Figure S4b inset). The pressure distribution of air on the sample shown in Figure S4b was calculated by an approach based on the Test Particle Monte Carlo (TPMC) method [Vinzenz Friedli. *Focused Electron- and Ion-Beam Induced Processes : In Situ Monitoring, Analysis and Modeling*. PhD thesis, 2008]. The nozzle was held by a Kleindiek Encoded Micromanipulator MM3E for fast and precise positioning and hence pressure control. If not specified otherwise, the shutter between the electron gun and the sample was only open for taking images during the experiments to minimize any electron beam effect to the NPs. The interval between two consequent images was at least 30 seconds.

SEM image processing. For the image sequence of heating Mg NPs from 350 – 490 °C, the brightness and contrast of all images were aligned to avoid the effect of brightness and contrast variations to the grayscale levels of the NPs. The method is as follows:

1. Segment the NPs and the substrate (i.e. background) by using the GrabCut method in OpenCV
2. Use Gaussian distribution to fit the distribution of the grayscale of the background
3. Determine the brightness and contrast adjustment factors from the fitted Gaussian distribution (corresponding to the mean and deviation of the distribution, respectively), and then apply the factors to the original images.
4. Use the GrabCut method to segment the NPs from the brightness and contrast –aligned images.

High-angle annular dark field scanning transmission electron microscopy (HAADF-STEM) images, STEM energy dispersive X-ray spectroscopy (STEM-EDS) maps and STEM electron energy loss spectroscopy (STEM-EELS) maps of samples drop cast on a Silicon nitride membrane were acquired at 200 kV on a FEI Osiris STEM with a Bruker Super-X quadruple EDS detector and a Gatan Enfinium ER 977 electron spectrometer STEM-EELS maps of the Mg bulk plasmon (Fig. 1h, S2) were produced by integrating the intensity of the spectrum image from 9.5 to 11.5 eV, and STEM-EDS maps (Fig. 1f) were obtained by integrating the Kα peaks of O and Mg.

Thermogravimetric Analysis (TGA) was performed using SDT 650 (TA Instruments) thermal analyzer. In typical experiments, 5mg of Mg-MgO NPs powder was heated in air atmosphere from room temperature to 550°C (15°C/min) and kept at this temperature for 20min.

X-Ray powder electron diffraction (XRD) was performed on a Bruker D8 DAVINCI with position sensitive detector (LynxEye EX) in coupled theta/2theta mode with a scan range of 30–70° and time per step of 0.68 seconds (0.01° per step). The source is Cu Kα, and samples were drop cast onto silicon wafers as a low-background holders. *In situ* high temperature XRD data were collected on Malvern-PanAlytical Empyrean 4-circle diffractometer, equipped with: a hybrid mirror/monochromator for parallel Kα1 monochromatic radiation; a PIXCel position sensitive detector; an Anton domed hot stage DHS 1100 for in-situ high temperature measurements. The analysis was performed in XRD characterization chamber in air atmosphere from room temperature to 450°C (10°C/min), and diffraction data was acquired every 50°C. The sample was maintained under isothermal conditions for 20min prior each XRD analysis.

Figures

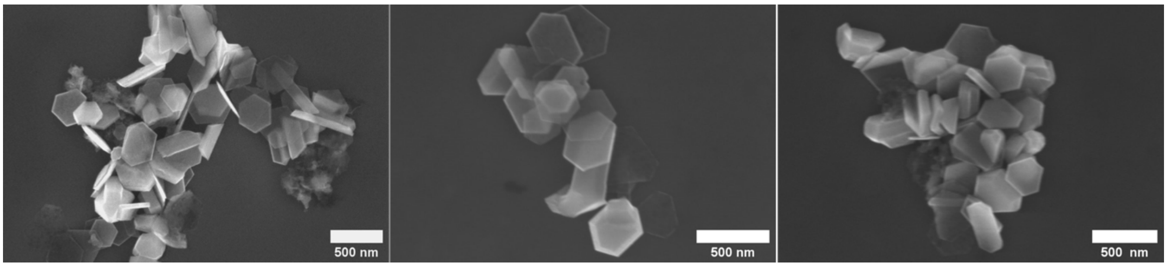

Figure S1. Additional SEM images of as-prepared Mg NPs.

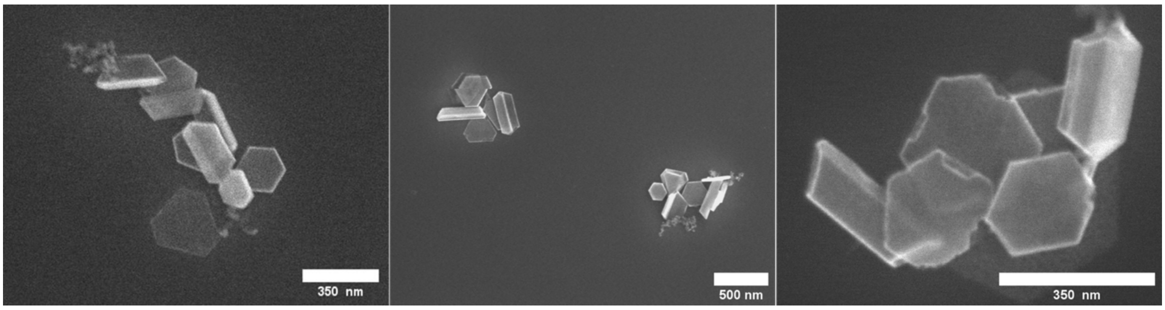

Figure S2. SEM images of Mg NPs after plasma-treatment.

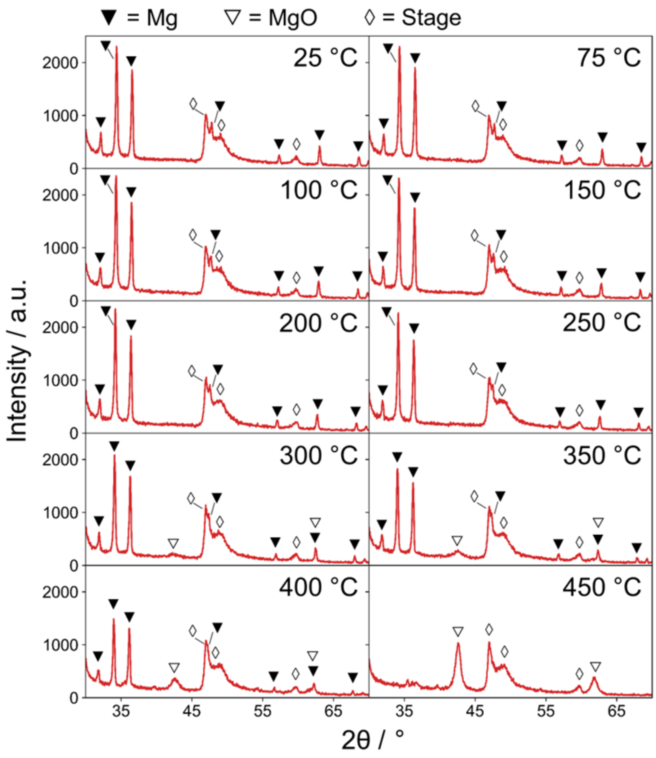

Figure S3. Additional XRD spectra of Mg NPs acquired at different temperatures in air atmosphere.

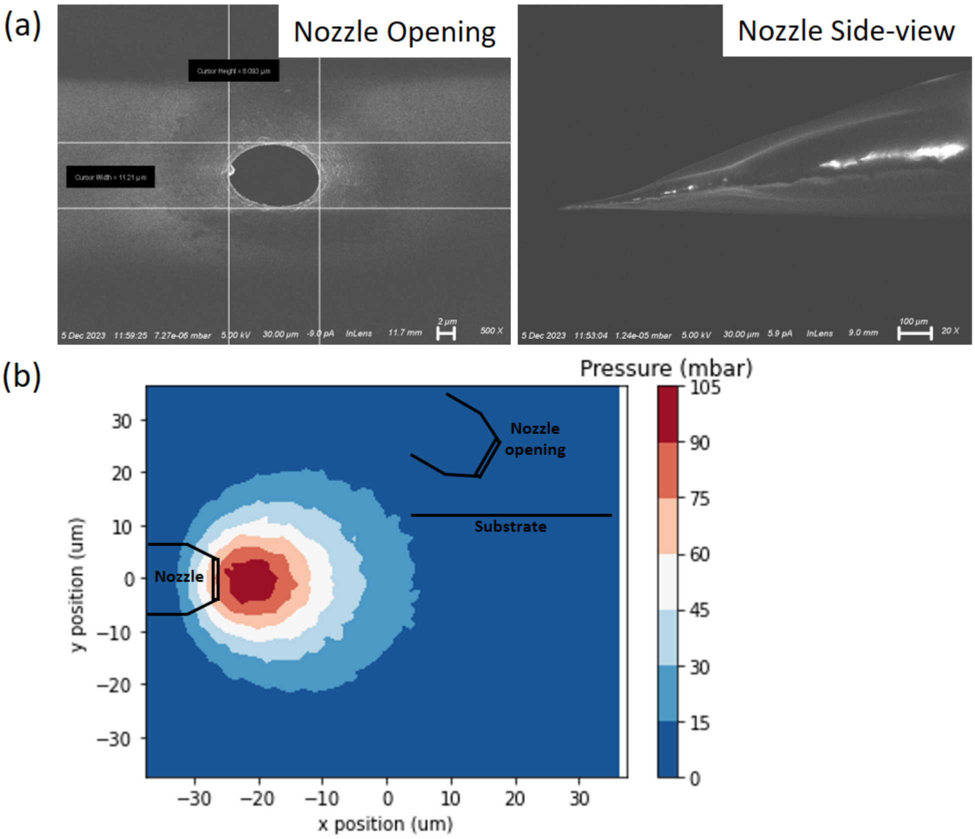

Figure S4. Gas injection system (GIS) in the *in-situ* SEM. (a) SEM images of the top and side view of the quartz nozzle; (b) Simulation of the air pressure distribution on the sample with the GIS set up (inset figure) at 300°C.

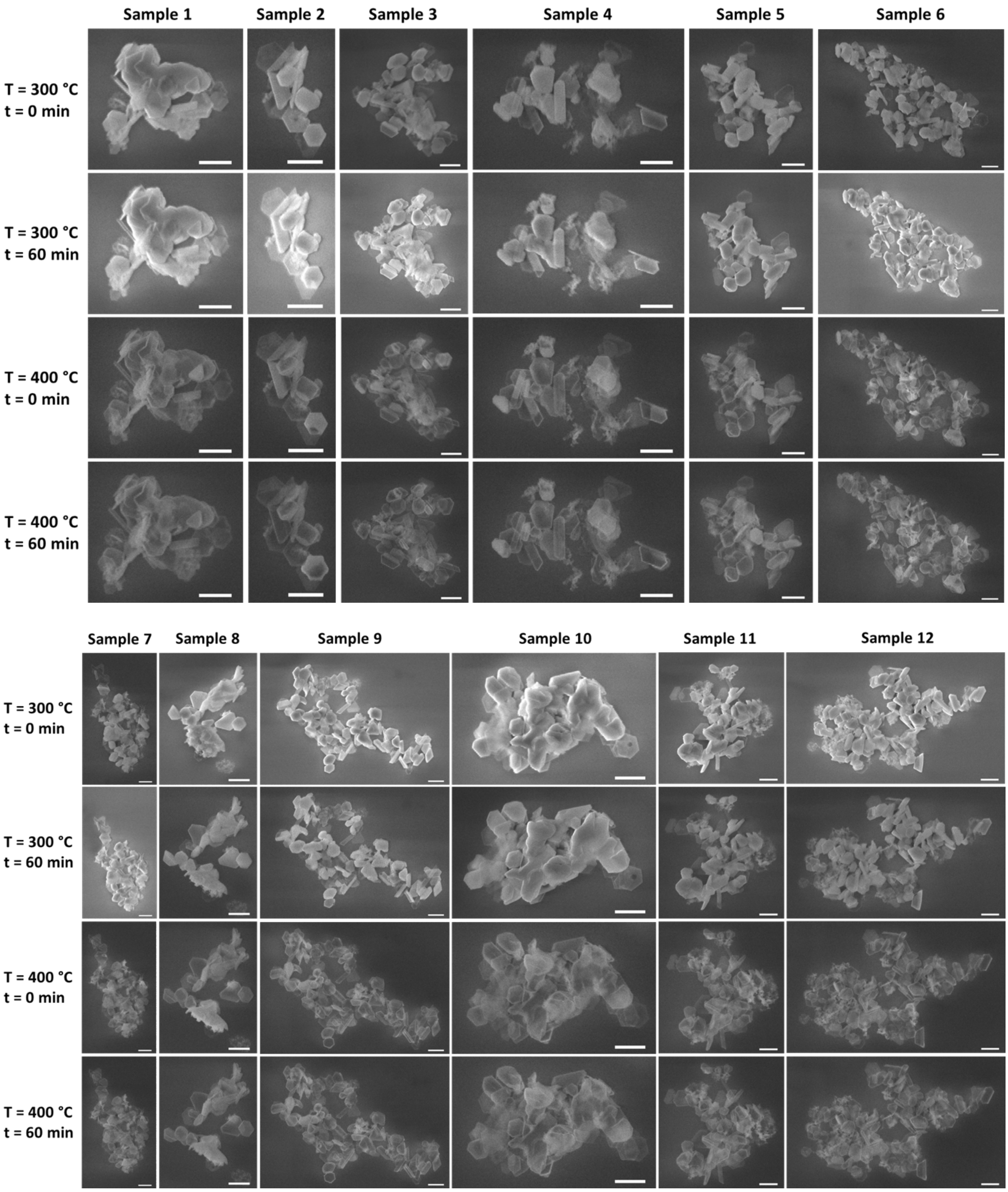
Sample 1
Sample 2
Sample 3
Sample 4
Sample 5
Sample 6
T = 300 °C
t = 0 min
T = 300 °C
t = 60 min
T = 400 °C
t = 0 min
T = 400 °C
t = 60 min
Sample 7
Sample 8
Sample 9
Sample 10
Sample 11
Sample 12
T = 300 °C
t = 0 min
T = 300 °C
t = 60 min
T = 400 °C
t = 0 min
T = 400 °C
t = 60 min

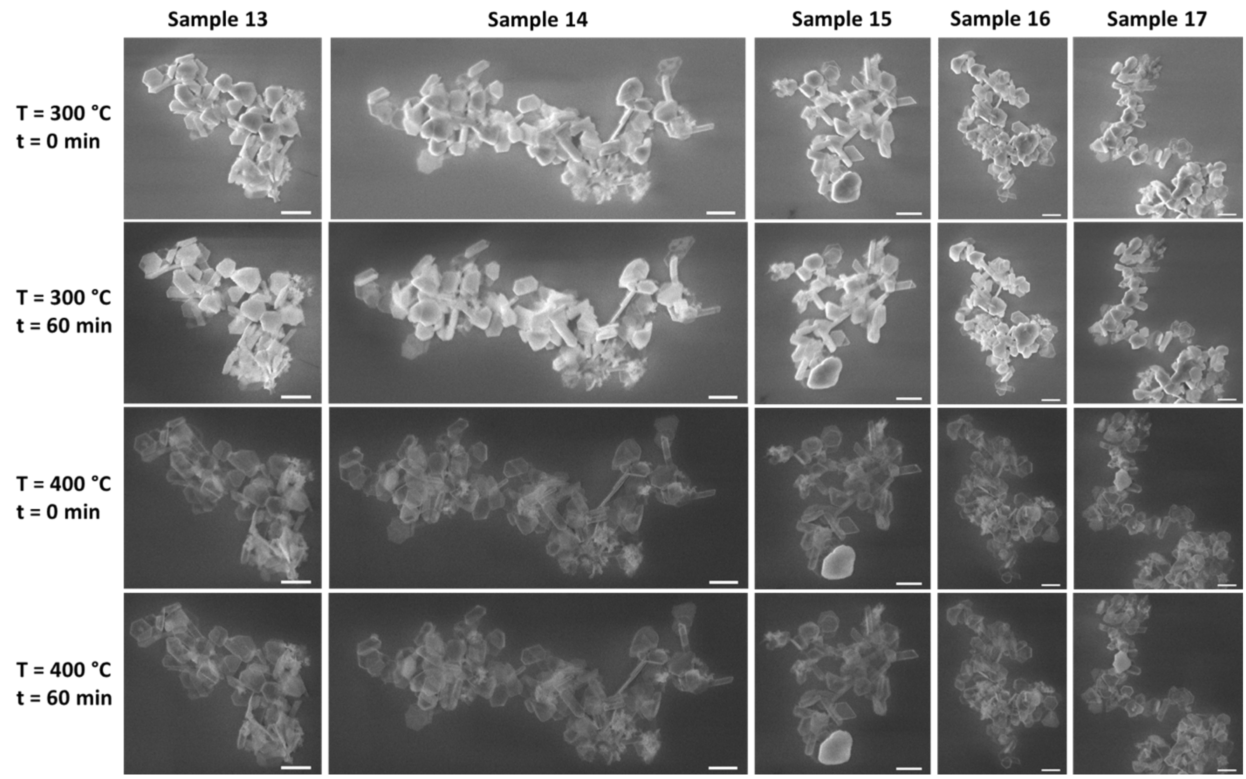

Figure S5. Additional *in-situ* SEM images of Mg-MgO NPs exposed to ~100 mbar of air at 300°C and 400°C taken after 0 and 60 min of air exposure. Scale bars, 500 nm.

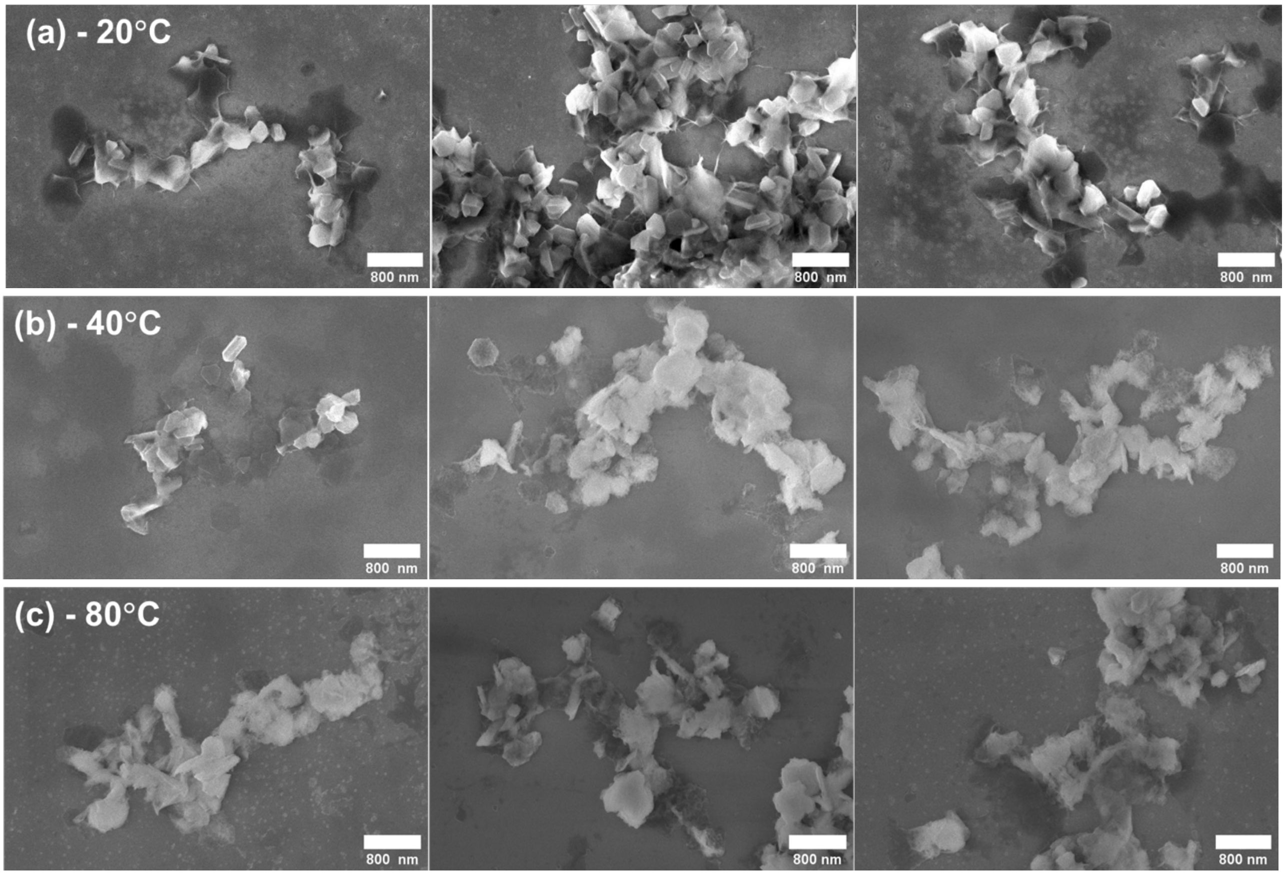

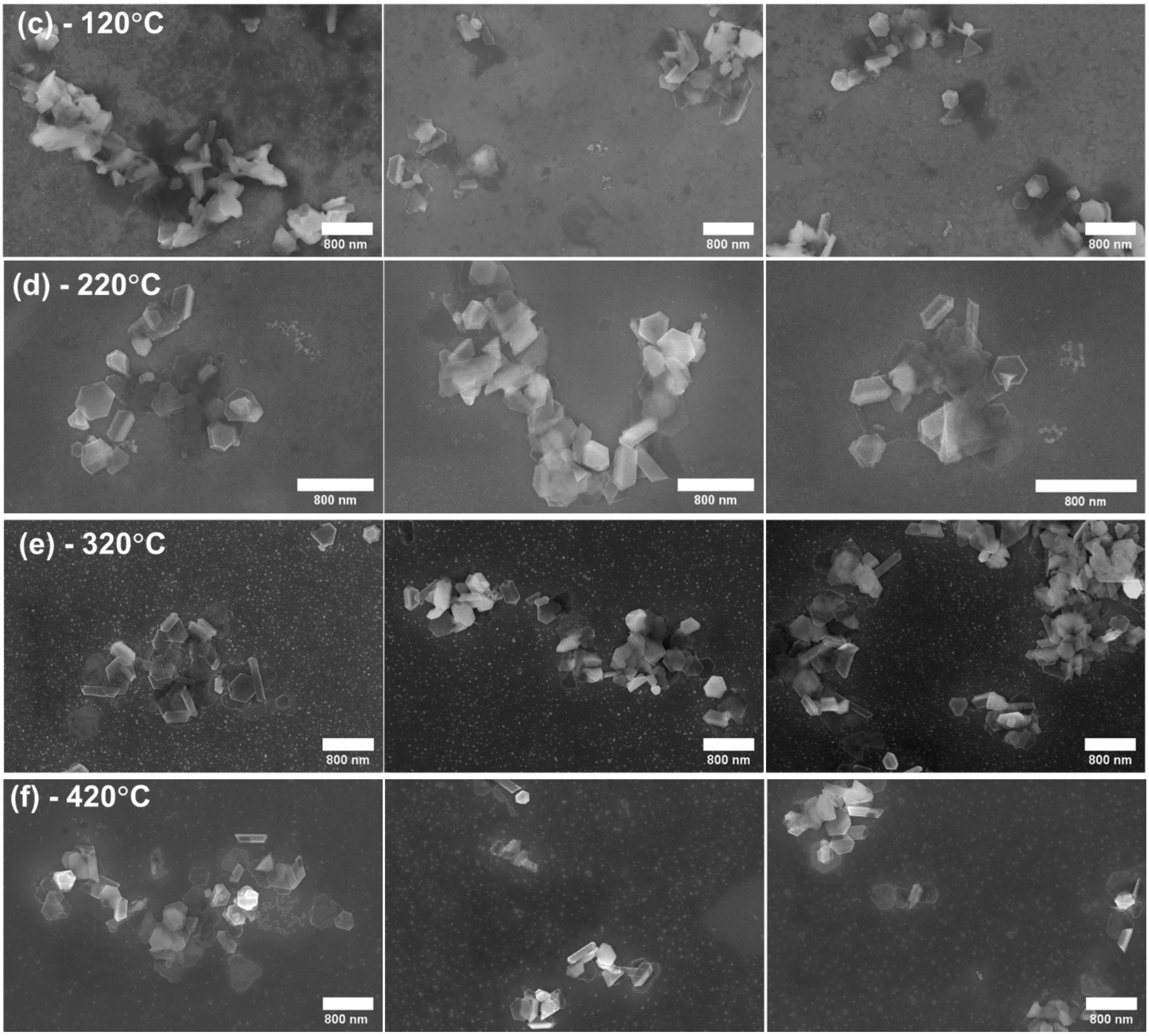

Figure S6. Additional SEM images of Mg NPs after treatment with 3.5 vol.% water vapor in nitrogen at different temperatures.

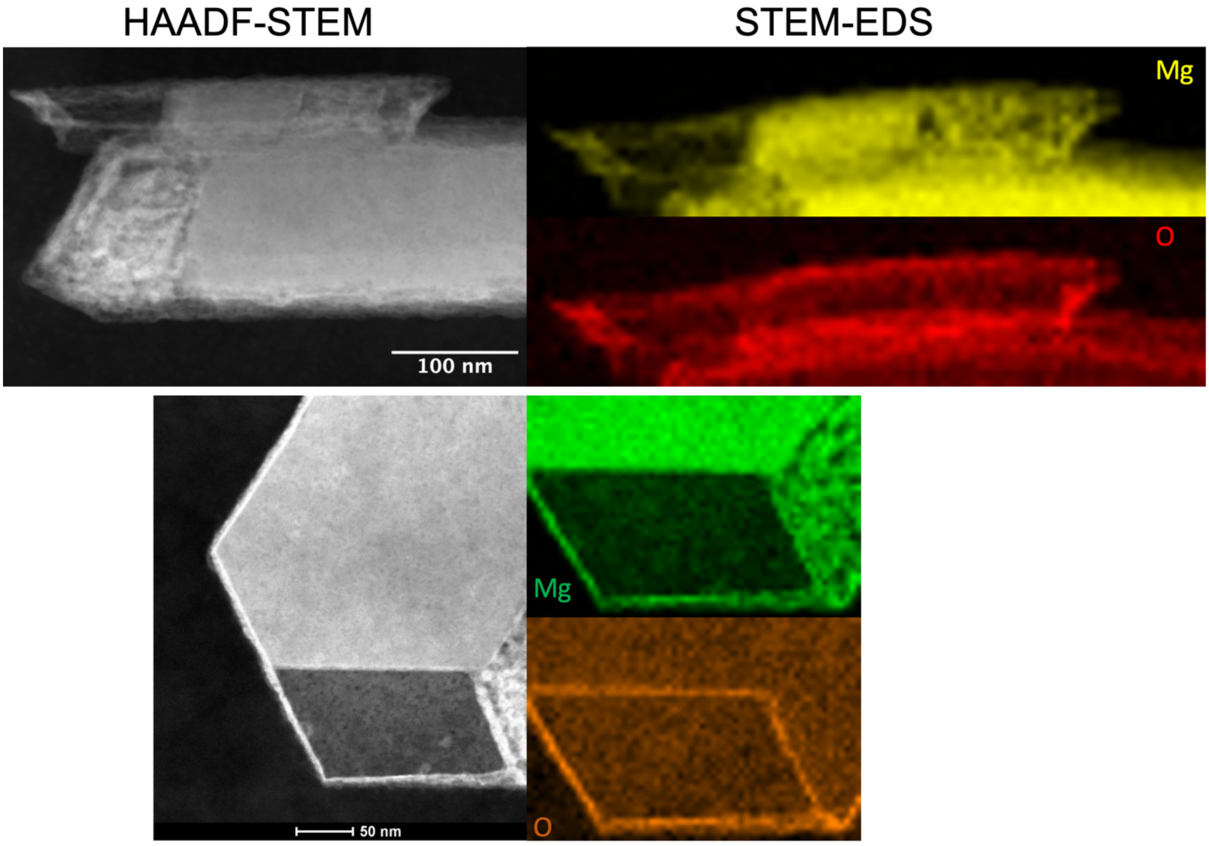

Figure S7. Additional STEM-EDS data. (left) HAADF-STEM images and (right) associated STEM-EDS maps of Mg and O of an Mg-MgO NP treated with 3.5 vol.% of H_2O in N_2 for 1h at 420°C.
